# Sexual Dimorphism in the Initial Apoptotic Switch During MASH Progression in Mice

**DOI:** 10.3390/ijms27031501

**Published:** 2026-02-03

**Authors:** Pradeep K. Rajan, Jacqueline A. Sanabria, Mathew S. Schade, Utibe-Abasi S. Udoh, Alexei Gorka, Sodhi Komal, Sandrine V. Pierre, Juan Sanabria

**Affiliations:** 1 Department of Surgery, Joan C. Edwards School of Medicine, Marshall University Huntington, Huntington, WV 25701, USA; 2 Marshall Institute for Interdisciplinary Research (MIIR), Huntington, WV 25701, USA; 3 Department of Nutrition, School of Medicine, Case Western Reserve University, Cleveland, OH 44106, USA

**Keywords:** apoptosis, senescence, oxidative species, diet modification, metabolism, MASH, HCC

## Abstract

MASH is a progressive liver disease closely associated with cellular senescence, which is present in more than 80% of hepatocytes in patients who develop hepatocellular carcinoma (HCC). Although MASH affects both sexes, the incidence of MASH-related HCC is two to four times higher in males. Our group has previously described two apoptotic switches during MASH progression and HCC development, implicating the ATP1A1 signalosome in the late switch. Here, we investigated the role of ATP1A1 and sex-specific differences in the early apoptotic switch during preclinical MASH progression. Male and female C57BL/6J mice (7 weeks old) were fed normal mouse chow (NMC) or a high-fat diet (HFD) for 12, 24, or 48 weeks (n = 5/sex/group). Total body weight (TBW) and body composition were assessed by serial measurement and echo-MRI. Plasma was analyzed by non-targeted metabolomics and glutathione profiling using LC-MS/MS. NAFLD activity scores (NAS), hepatic senescence, and apoptosis were quantified in liver tissue. Statistical analyses were performed using GraphPad Prism and R. Males gained greater TBW and lean and fat mass than females (*p* < 0.05). At 24 W, males demonstrated higher GSH:GSSG ratios and lower ophthalmate levels than females (*p* < 0.05), consistent with altered redox balance. HFD-fed females showed increased succinic and deoxycholic acid levels, whereas males exhibited higher butyric acid levels across all time points (*p* < 0.05). Males had a higher mTOR 1 expression at 24 W and P53 at 12 W compared to females on HFD, but a lower Grb2 expression at 24 W (*p* < 0.05). By 24 W, males had lower fibrosis scores and reduced apoptotic activity compared with females (*p* < 0.05), despite similar levels of cellular senescence. The expression of ATP1A1, survivin, and SMAC did not differ by sex or diet, although an upregulation trend in both ATP1A1 and survivin was noted in the male-HFD group. There is sexual dimorphism in the response to HFD during the transition from senescence to the apoptosis-first apoptotic switch in MASH progression.

## 1. Introduction

The global incidence and prevalence of chronic liver disease and its major consequences—end-stage liver disease (ESLD) and hepatocellular carcinoma (HCC)—continue to rise, driven historically by viral hepatitis and increasingly by metabolic dysfunction associated with the obesity epidemic [1,2]. Between 2019 and 2022, deaths attributable to viral hepatitis increased from 1.1 to 1.3 million worldwide, and by 2024, viral hepatitis had become the second leading infectious cause of mortality globally. Nonetheless, the overall burden of viral hepatitis-related liver disease is expected to decline because of the widespread availability of highly effective antiviral therapies [3,4,5]. In contrast, the prevalence of obesity continues to accelerate, with an estimated 2.2 billion individuals projected to be overweight and 1.1 billion to be obese by 2030. Metabolic dysfunction–associated steatohepatitis (MASH)–related ESLD is now the second most common indication for liver transplantation in the United States [6,7]. MASH disproportionately affects racial and ethnic minorities—including African American and Hispanic populations—as well as individuals from lower socioeconomic backgrounds, reflecting persistent disparities in healthcare access. Moreover, the incidence of MASH and MASH-related HCC is substantially higher in males than in females, with an estimated male-to-female ratio of approximately 2–4:1 [8]. Similar disparities contribute to poor outcomes and reduced survival in HCC, a malignancy with one of the fastest-rising incidence rates and the highest cancer-related mortality in the United States [9,10,11,12,13].

Investigating the role of Src kinase (Src) in ATP1A1 signaling, we observed both in vitro and in vivo upregulation of survivin (anti-apoptotic) and downregulation of Smac/DIABLO (SMAC; pro-apoptotic) during the progression of MASH to HCC. These alterations, driven by epigenetic modifications, suppressed autophagy and triggered a critical apoptotic switch through activation of the PI3K → Akt pathway, accompanied by nuclear extrusion of FoxO3. This signaling cascade promoted metabolic dysregulation, enhanced hepatocyte proliferation, and initiated primary liver carcinogenesis [14]. Based on these findings, we defined two critical apoptotic switches: while a high ratio of SMAC/survivin pushes hepatocytes from an accelerated senescence state into apoptosis (first switch), a low ratio drives cells into uncontrolled division (second switch). Importantly, this survivin/SMAC dysregulation signature was also detected in liver samples from patients with HCC, but not in individuals with normal livers. Senescence develops through the activation of multiple pathways in response to stress stimuli, [14] including DNA damage (from radiation, oxidative stress, or telomere dysfunction), [15,16,17] metabolic derangements such as mitochondrial dysfunction characteristic of MASH, and proinflammatory signaling driven by autophagic dysregulation [18,19,20,21,22]. Insulin resistance further exacerbates cellular metabolic dysregulation [16,17]. Apoptotic activity correlates with MASH severity, and the timing and magnitude of the apoptotic switch are associated with the development of MASH-HCC [22,23]. Thus, progression from hepatocellular senescence to apoptosis represents a critical inflection point in MASH pathogenesis.

Sex disparities in obesity influence MASH progression, and both disease severity and MASH-related complications show sex-specific patterns. Preclinical evidence demonstrates that mice exposed to a high-fat diet (HFD) develop white adipose tissue (WAT) senescence within two weeks, marked by increased senescence-associated β-galactosidase (SA-β-Gal) and expression of cyclin-dependent kinase inhibitors 1A and 2A [24,25]. We hypothesized that sex differences in the early apoptotic switch may shape MASH progression, where the role of the AT1A1 signalosome may be limited to alterations in cell autophagy [26]. In this preclinical study, females exhibited similar levels of hepatocellular senescence compared with males, but showed significantly higher apoptotic activity during MASH. These findings align with the well-recognized predisposition of males to develop MASH-related HCC [17].

## 2. Results

### 2.1. Effect on Total Body Compartments by Diet/Sex in the MASH Mice Model

**Figure 1:**
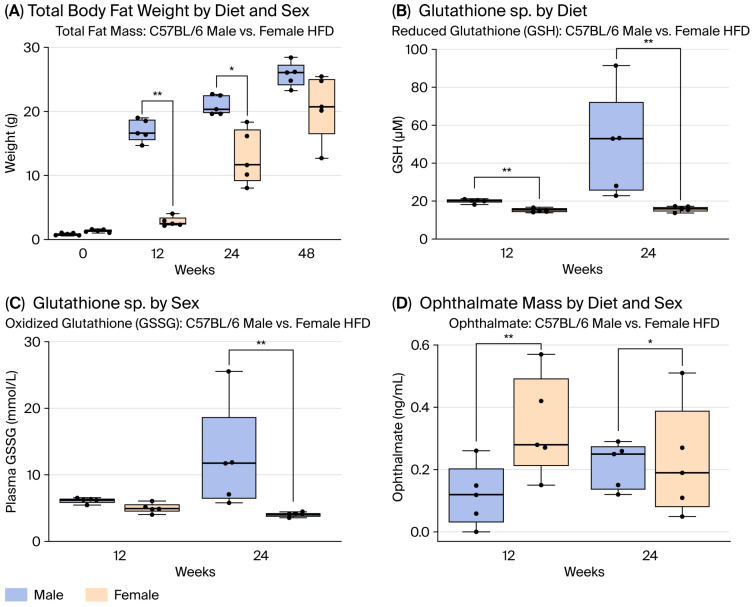
Effects of diet and sex on body composition and redox status in the MASH mouse model. (**A**) Both male and female mice exposed to HFD showed consistent increases in TBW; however, HFD males gained significantly larger lean and fat mass than females at 24 W. (**B**,**C**) Significant group- and time-dependent differences were observed in reduced (GSH) and oxidized (GSSG) glutathione. At 24 W, HFD-fed males had higher GSH and GSH:GSSG ratios than females. (**D**) Ophthalmate levels were elevated in HFD-fed mice of both sexes compared with the NMC controls. At 12 W, a diet-specific difference was evident in females. Male-NMC mice had lower ophthalmate levels than female-NMC at 0 W, and male-HFD mice had lower levels than female-HFD at 12 W and 24 W (* *p* < 0.05; ** *p* < 0.01 by ANOVA with Tukey’s post hoc test or *t*-test; n = 5).

Male mice have higher total body weight (TBW) than female mice in both the NMC and HFD groups (Figure S1). The male-HFD group has higher total fat mass compared to the female-HFD group at 12 and 24 W (Figure 1A). When we standardized the fat mass gain (FAT/TBW), the gain of fat mass remained significantly different in mice under HFD by sex (mean of 3.1 for females vs. 2.6 for males) at 24 W. These findings highlight important sex differences in body composition in response to dietary changes.

### 2.2. Cellular Redox Assessment by Diet/Sex in the MASH Mice Model

The male-HFD group had increased GSH compared to females by diet (Figure S2). The male-HFD and female-HFD groups had increased GSH and GS:SG compared to the male/female-NMC groups at 24 W. In addition, male-HFD displayed higher GSH and GS:SG levels at 24 W compared to female-HFD (Figure 1B,C). Ophthalmate (OA) was higher in male/female-HFD vs. male/female-NMC at 24 W (Figure S3). Male-HFD had lower levels of OA than female-HFD at 12 and 24 W (Figure 1D). The cell-redox response differs by sex and diet.

### 2.3. Metabolic Response by Diet/Sex in the MASH Mice Model

**Figure 2:**
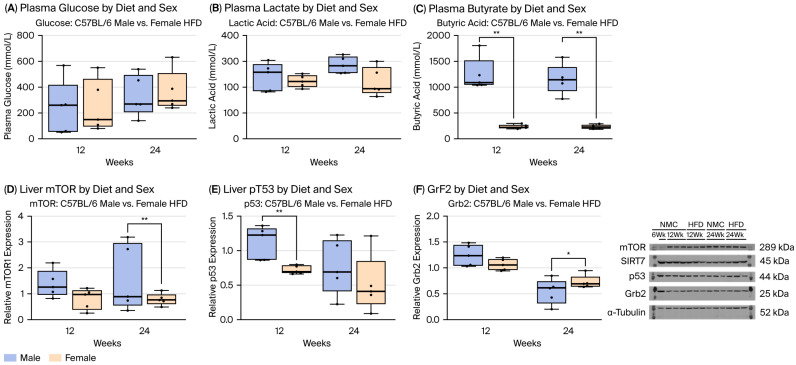
Metabolite profiles and protein expression in the MASH mouse model by diet and sex. (**A**) In HFD-fed mice, males and females showed similarly elevated blood glucose levels. (**B**) Plasma lactate did not differ between the male- and female-HFD groups. (**C**) In contrast, butyrate levels differed significantly by sex under HFD; males had higher concentrations than females at all time points (* *p* < 0.05, ** *p* < 0.01). (**D**) mTOR1 expression increased progressively with HFD, with males showing significantly higher levels than females at 24 W. (**E**) p53 expression rose significantly in HFD-fed males at 12 W compared with females. (**F**) Although time-dependent changes were minimal, Grb2 expression was significantly lower in HFD-fed males at 24 W. Representative Western blots illustrate hepatic protein levels for males and females at 0, 12, and 24 W. Statistical analyses were performed using two-way ANOVA with Tukey’s post hoc test, followed by *t*-tests between the groups to assess the effects of diet, time, and sex; or Kruskal–Wallis test with Benjamini–Hochberg correction, and group comparisons were performed using the chi-square (χ^2^) test (* *p* < 0.05, ** *p* < 0.01; n = 5).

HFD males showed significantly higher glucose and lactic acid levels than those on an NMC at 24 W (Figure S4A,B). The BS and lactic levels of HFD males were not different compared to HFD females. In addition, there was a significant difference in butyric acid in the HFD males and females at all time points, where the male groups showed a significantly higher butyric acid level compared to females (Figure 2C and Figure S5). Although there were no differences in BS and lactate levels by sex in the HFD group, there were significant differences in butyric acid (a surrogate for β-lipid oxidation) at all time points by sex in the HFD group.

### 2.4. Protein Expressions

To evaluate the effects of a high-fat diet (HFD) on signaling molecules involved in metabolism and cellular stress responses, we quantified hepatic protein levels of mTOR1, SIRT7, p53, and Grb2 in male and female C57BL/6J mice (Figure S6). At 24 W, males exhibited significantly higher mTOR1 expression, and at 12 W higher p53 expression, compared with HFD-fed females (Figure 2D–E, *p* < 0.01), while demonstrating lower Grb2 expression at 24 W (Figure 2F, *p* < 0.05). SIRT7 expression did not differ by sex or diet. We also assessed proteins implicated in the apoptotic switch toward malignancy—survivin, SMAC, and ATP1A1. No significant sex- or diet-dependent differences were observed at 12 or 24 W in HFD-fed mice (Figure S7, *p* > 0.05). However, there was a trend toward increased ATP1A1 expression in both sexes under HFD and a trend toward survivin upregulation in HFD-fed males. Overall, metabolic signaling proteins (mTOR1, p53, and Grb2) showed clear sex- and diet-dependent regulation, whereas expression of apoptotic modulators (survivin, SMAC) and ATP1A1 was not significantly altered during the early switch in MASH progression.

### 2.5. Non-Targeted Metabolomics by HPLC-MS/MS

**Figure 3:**
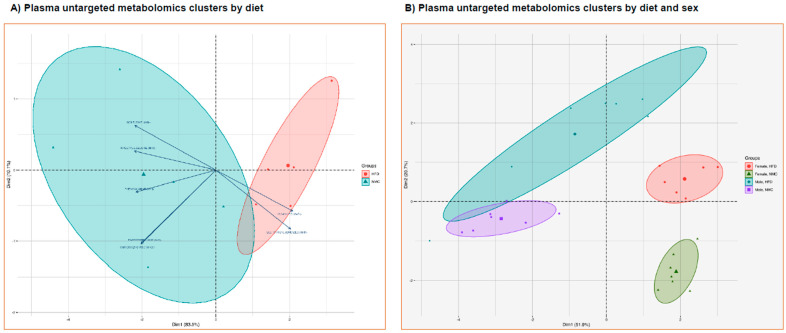
Effect on non-targeted metabolites by diet and sex in the MASH mice model. (**A**) Metabolites detected in plasma samples showed distinct clustering by diet group (NMC in pink and HFD in magenta), indicating significant metabolic differences between the NMC and HFD groups. (**B**) Additionally, notable biochemical changes were observed over the 24-week period in the male groups (NMC in purple and HFD in magenta) compared to the female groups (NMC in green and HFD in pink). Over the 24 W period, male mice displayed more pronounced biochemical shifts than females.

HFD-induced female mice showed an increased level of succinic acid and deoxycholic acid at 24 W (Figure S8). In the NMC groups, males exhibited higher levels of deoxycholic acid at 24 W compared to females; however, there was no significant difference in the HFD groups for either gender. The characteristics of the matching conditions of metabolites were visualized and analyzed by principal component analysis (PCA) to identify actual clusters. Through the analysis, significant differences in metabolic profiles were found between the NMC and HFD groups (Figure 3A). There was a significant difference between the NMC group and the HFD group, where significant biochemical changes were observed in the male vs. female groups at 24 W (Figure 3B). There were significant differences in the metabolic signature response to HFD based on sex and diet.

### 2.6. Liver Morphology

**Figure 4:**
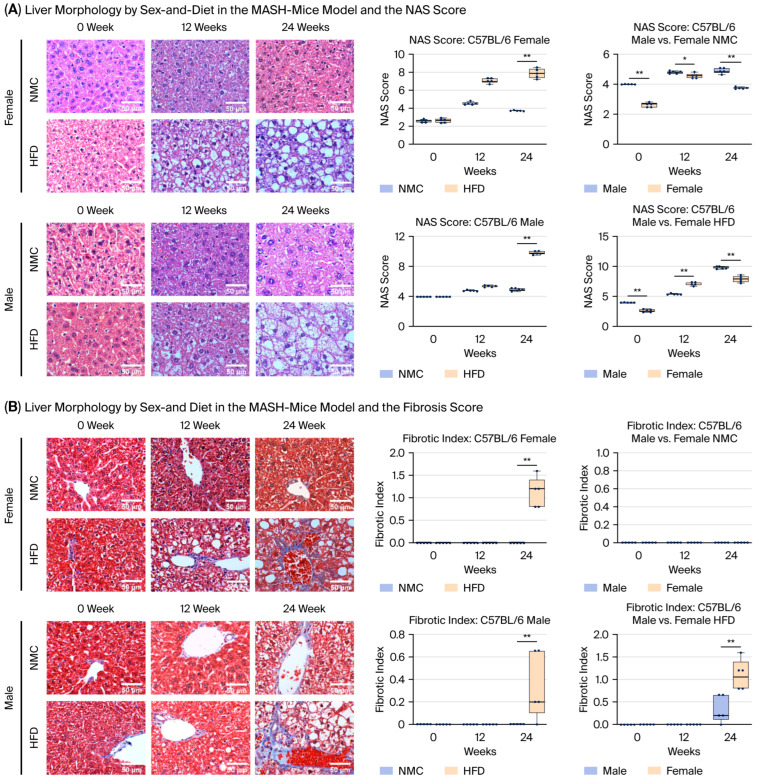

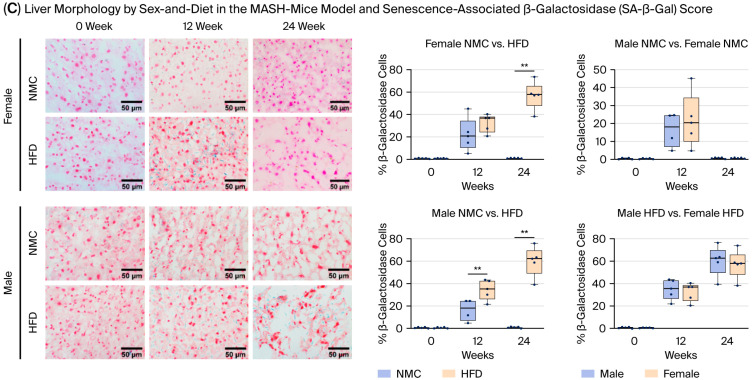
Liver histological changes by diet and sex in the MASH mouse model. (**A**) Steatosis and inflammation: HFD-fed mice of both sexes developed the characteristic MASH phenotype, including steatosis, inflammatory infiltration, and ballooning. NAS scores corroborated these findings. Although HFD-fed males exhibited lower NAS at 12 W, they progressed to higher scores than females by 24 W. (**B**) Fibrosis: Collagen deposition was greater in the HFD groups compared with the NMC controls. Under HFD, females displayed significantly more fibrosis than males, whereas no sex differences were observed in the NMC groups. (**C**) Cellular senescence: Both sexes showed an age-related increase in senescent cells, most prominently in HFD-fed mice. Senescence increased as early as 12 W under HFD, whereas the NMC groups showed no further accumulation beyond baseline. HFD-fed mice demonstrated a significant rise in SA-β-gal-positive cells, with no sex differences. Images were captured at 20×/40× magnification (** *p* < 0.01; Kruskal–Wallis test with Benjamini–Hochberg correction; group comparisons were performed using the chi-square (χ^2^) test (* *p* < 0.05, ** *p* < 0.01; n = 5)).

**Figure 5:**
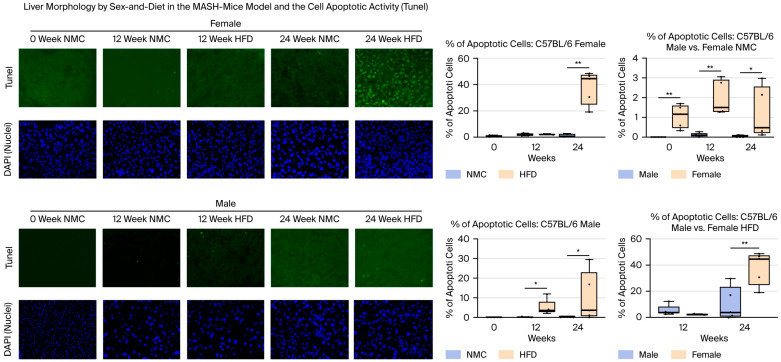
TUNEL staining of apoptotic activity in the MASH mouse model. Apoptosis was quantified as the percentage of TUNEL-positive cells relative to the total number of hepatocytes. Representative TUNEL staining (green) is shown at 0, 12, and 24 weeks (20×/40× magnification). Apoptotic activity was significantly higher in HFD-fed mice of both sexes at 24 W compared with NMC controls. Notably, the increase was more pronounced in females than in males under HFD conditions (** *p* < 0.01, * *p* < 0.05 by Kruskal–Walli’s test with Benjamini–Hochberg correction; group comparisons were performed using the chi-square (χ^2^) test (* *p* < 0.05, ** *p* < 0.01; n = 5)).

The NAS score was significantly higher in the male/female-HFD groups compared to the NMC groups at 24 W. Male-NMC/HFD had higher NAS scores compared to female-NMC/HFD at all time points (Figure 4A,B). The liver fibrotic index was higher in male/female-HFD compared to the male/female-NMC groups at 24 W. The fibrotic index was similar in the NMC group. In contrast, females on an HFD had a higher fibrotic index than males on HFD at 24 W (Figure 4C). The increase in senescent cells was more prominent in the male and female groups on HFD, though it was not a trend seen in the NMC group. Male-HFD had a significant increase in β-gal expression than male-NMC at 12 and 24 W, while female-HFD had a significant increase in β-gal expression than female-NMC at 24 W. There was no difference in senescent cell activity between males and females with a similar diet (Figure 4A,B). Apoptotic activity was significantly higher in both male and female groups on an HFD after 24 W, compared to those on an NMC diet. More importantly, female groups on NMC/HFD exhibited higher apoptotic activity than male groups on the same diet (Figure 5). Although there were no differences between the activities of cell senescence among the females and males exposed to HFD, females exhibited a higher degree of fibrosis and an increased apoptotic response throughout MASH progression.

## 3. Discussion

Cellular senescence involves metabolic dysregulation and morphological changes that contribute to MASH pathogenesis, with documented sex-based differences in its incidence [27,28]. In cirrhotic livers with HCC, up to 80% of parenchymal cells display senescent features [29,30]. In this study, we observed a clear sexual dimorphism in response to HFD, influencing multiple pathways relevant to the early transition from senescence to apoptosis during MASH progression. Sex- and diet-dependent differences were identified in redox balance (glutathione sp.), metabolic function (insulin resistance, β-lipid oxidation), metabolic protein expression (mTOR1, p53, Grb2), senescence and apoptotic activity, and liver morphology (NAS and fibrosis scores). Relative to HFD-fed females, HFD-fed males exhibited increased TBW (lean mass), lower cell redox (higher GSSG and lower ophthalmate), higher β-lipid oxidation with comparable insulin resistance, and distinct plasma metabolomic signatures. In addition, HFD-fed males showed increased mTOR1 and p53 expression, higher NAS scores, lower fibrosis scores, lower apoptotic activity, and an upward trend in ATP1A1 and survivin expression. Consistent with prior reports of sex-dependent differences in obesity-related liver disease [25], our findings demonstrate that sex and diet interact to modulate metabolic and cellular dysregulation during senescence-associated MASH progression.

The incidence of liver disease continues to rise across North America, driven largely by metabolic disturbances associated with obesity and insulin resistance. A hallmark of chronic liver injury is hepatocyte cell cycle arrest—present in more than 80% of affected cells—whose transition to apoptosis is tightly linked to MASH progression. Under the low-redox conditions characteristic of MASH, the ATP1A1-dependent ROS feedforward loop becomes chronically activated. Beyond its metabolic role, ATP1A1 functions as a tumor suppressor through survivin/SMAC-mediated feedback. Notably, SMAC and survivin expression patterns in human HCC specimens differ markedly from those in healthy livers, MASH patients, and individuals with hepatic metastases [14]. Consistent with this model, restoration of ATP1A1 signaling reverses epigenetic alterations and re-establishes autophagic flux—early events in MASH progression that are critical for preventing malignant transformation [26]. In this study, we observed a clear sex-based difference in apoptotic activity; however, no significant sex- or diet-dependent changes were detected in hepatic ATP1A1, survivin, or SMAC expression. Importantly, although ATP1A1 protein levels remained stable during the early apoptotic switch, Na^+^/K^+^-ATPase function declined significantly, activating ATP1A1 signaling and signaling reduced autophagic activity and protein renewal [26]. These findings likely reflect the early disease time points examined, which may be insufficient for detectable ATP1A1 pathway re-programming, despite the trend toward increased survivin expression in males. Alternatively, sex differences could arise from distinct hormonal milieus, even in the context of similar protein expression profiles.

Male mice showed greater susceptibility to HFD-induced weight gain, with increases in total body weight and lean mass. Because males gained a more metabolically active lean mass, they may tolerate metabolic stress longer before undergoing an apoptotic switch. These patterns likely reflect sex-specific adipose distribution regulated by steroid hormone receptors [31,32]. Under metabolic stress, activation of the polyol pathway depletes NADPH and reduces glutathione reductase (GR) activity, lowering reduced glutathione (GSH) levels [33]. Diminished GSH weakens antioxidant defenses, elevates reactive oxygen intermediates (ROI), and promotes oxidative injury—an established mechanism underlying hyperglycemia-associated metabolic dysfunction [34]. In this study, we observed clear sex- and diet-dependent differences in glutathione species and ophthalmate, along with alterations in circulating glucose, a surrogate of insulin resistance [35,36]. Despite the liver’s central role in lactate clearance, no sex-based differences in plasma glucose or lactate were detected within the HFD groups. β-lipid oxidation increased in a sex- and diet-dependent manner, as reflected by changes in key metabolic intermediates. Excess lipid load may exceed skeletal muscle oxidative capacity, leading to intracellular lipid accumulation and insulin resistance [37]. β-oxidation was assessed through plasma butyrate levels [38,39]. Butyrate promotes fatty acid oxidation and suppresses lipogenesis, [40,41] and we observed significant sex-dependent differences across all HFD time points. Prior studies similarly show that butyrate enhances insulin sensitivity, reduces fat mass, and increases lean mass in male mice [42,43]. Consistent with previous reports, [44] succinic acid levels increased in HFD-fed mice, serving as a paracrine marker of hepatic injury; this effect was more pronounced in females. Elevated butyrate may reflect altered mitochondrial substrate utilization and β-oxidative capacity under lipotoxic stress, consistent with metabolic inflexibility during MASH progression. These metabolic differences may contribute to the sex-dependent amplification of inflammatory cascades and apoptotic susceptibility during MASH progression. Given the central role of the ATP1A1 Na^+^/K^+^-ATPase signalosome in coordinating redox balance, mitochondrial stress responses, and apoptosis, differential butyrate profiles may intersect with ATP1A1-mediated signaling to shape divergent inflammatory and apoptotic trajectories between the sexes under HFD. Although males showed no significant change, HFD-fed females exhibited a marked rise in deoxycholic acid, a bile acid associated with high-fat diet-induced inflammation and progressive liver injury [45].

Male mice demonstrated progressive upregulation of mTOR1 and p53 under HFD, reflecting enhanced nutrient-sensing and silencing of tumor suppression. Male-specific induction of mTOR may delay the transition from senescence to apoptosis, contributing to earlier hepatocyte loss and tissue remodeling in females [46,47]. Conversely, mTOR inhibition is associated with autophagy induction and SASP activation—processes implicated in hepatocellular injury and fibrogenesis in MASH [48,49]. Thus, the relative preservation of mTOR signaling in females may provide protection by maintaining anabolic balance and mitigating catabolic stress. The rise in p53 in males most likely represents a compensatory response to oxidative stress, DNA damage, and lipotoxic injury—core features of MASH. p53 activation promotes transcription of pro-apoptotic genes (Bax, PUMA) and cell-cycle inhibitors (p21), thereby enhancing apoptosis and senescence, both central components of MASH progression [50,51]. More pronounced p53 activation in males may indicate stronger stress response signaling, contributing to sex-dependent differences in disease course or to counter-regulatory mechanisms designed to limit further hepatic injury. These findings align with reports of attenuated or delayed MASH progression in females, which is potentially mediated by estrogen signaling and improved mitochondrial resilience [52,53]. The distinct p53 pattern in males may also allow for the maintenance of a senescent state, delaying or preventing the early apoptotic switch observed in females. Although sex differences in SIRT7 expression were less pronounced than expected, modest suppression in females may still influence mitochondrial performance and chromatin structure. SIRT7 deacetylates p53, stabilizing its activity and modulating senescence and apoptosis pathways [54], while reduced SIRT7 expression has been linked to impaired mitochondrial biogenesis, diminished DNA repair capacity, and enhanced senescence. Grb2 further integrates insulin and growth factor signaling through Ras/MAPK cascades. [55].

ATP1A1 (Na^+^/K^+^-ATPase α1 subunit) functions as both an ion transporter and a signaling transducer that modulates redox balance, apoptosis, and fibrogenic pathways—key processes in MASH. The sex- and diet-dependent regulation of ATP1A1 observed here underscores its role in hepatic stress signaling during disease progression. In HFD-fed males, ATP1A1 expression increased from 12 to 24 W, whereas females maintained stable expression across both time points (Figure S7). Under normal chow, levels were comparable between the sexes. Notably, females exhibited higher ATP1A1 expression than males at 12 W on HFD, but this pattern reversed by 24 W. These divergent temporal patterns likely reflect distinct adaptive programs: an early compensatory rise in females may indicate heightened mitochondrial vulnerability and the initiation of premature apoptotic signaling, while delayed ATP1A1 upregulation in males may be associated with prolonged senescence and survival signaling. Given that ATP1A1 regulates survivin and redox homeostasis, these findings support its involvement in the sexually dimorphic apoptotic switch during MASH progression and highlight ATP1A1 as a potential target for sex-specific therapeutic intervention. Hepatocyte apoptosis in MASH is governed by a balance between mitochondrial pro-apoptotic mediators, such as Second Mitochondria-Derived Activator of Caspases (SMAC), and anti-apoptotic regulators, including survivin. Disruption of this equilibrium contributes to hepatocellular injury, inflammation, and potential malignant progression through apoptotic-switch mechanisms [56]. Survivin and SMAC are central to cell-cycle control, and alterations in their expression can promote cell-cycle arrest or apoptosis [23]. In this study, survivin expression did not differ significantly by sex or diet, though a modest upward trend was noted in HFD-fed males at 12 W (Figure S7), suggesting possible age-related induction with chronic metabolic and mitochondrial stress [57]. SMAC levels showed no significant changes with either sex, diet, or time point. SMAC facilitates apoptosis by promoting caspase activation through the cytochrome-c/Apaf-1/caspase-9 cascade, where it binds and neutralizes inhibitor of apoptosis proteins (IAPs), enabling activation of caspase-9 and downstream executioner caspases [58]. The absence of diet- or sex-dependent variation in SMAC expression suggests that mitochondrial apoptotic priming during early MASH progression may be less reliant on SMAC dynamics, with survivin-ATP1A1 regulatory mechanisms and other metabolic stress signals playing more prominent roles in the sexually dimorphic apoptotic shift.

Both male and female HFD-fed mice exhibited increased hepatocellular senescence and apoptosis, confirming the detrimental cellular effects of chronic HFD exposure. Because senescence is central to MASH progression, we assessed β-galactosidase activity as a marker of senescent cells [59]. HFD-driven increases in SA-β-gal-positive cells were consistent with prior studies conducted under similar conditions in adipose tissue [60]. Although the proportion of senescent hepatocytes at 24 W did not differ by sex, activity levels revealed a six-fold increase in males on HFD, whereas no change was observed in females. This divergence likely reflects the higher apoptotic activity seen in females at 24 W, which may limit the accumulation of surviving senescent cells. Taken together, these results demonstrate sex-specific metabolic responses to HFD: Males exhibit greater senescent cell activity and lower apoptotic activity, accompanied by higher β-lipid oxidation and comparable insulin resistance.

Programmed cell death by apoptosis is a fundamental tumor-suppressive mechanism that protects tissues from malignant transformation by eliminating cells harboring irreparable genomic, metabolic, or oncogenic damage [61,62,63]. Efficient activation of intrinsic (mitochondrial) and extrinsic apoptotic pathways prevents the clonal expansion of cells with DNA mutations, chromosomal instability, or aberrant proliferative signaling. Conversely, evasion of apoptosis is a hallmark of cancer and facilitates tumor initiation, progression, and therapeutic resistance. Defects in apoptotic signaling—such as dysregulation of BCL-2 family proteins, impaired mitochondrial outer membrane permeabilization, reduced caspase activation, or overexpression of inhibitors of apoptosis proteins (IAPs) like survivin—allow survival of damaged cells and promote oncogenesis. In chronic inflammatory and metabolic disease states, including metabolic dysfunction-associated steatohepatitis (MASH), persistent apoptotic resistance coupled with compensatory proliferation creates a permissive environment for malignant transformation. Thus, timely and regulated apoptosis serves as a critical barrier to cancer development, whereas its suppression shifts cellular fate toward senescence escape, genomic instability, and tumorigenesis.

The findings of these studies are limited by predominantly mechanistic, hypothesis-driven designs. Nevertheless, it is well documented that mitochondrial structure and function exhibit pronounced sex-specific differences that influence cellular metabolism, redox homeostasis, and susceptibility to disease [64,65,66,67,68]. In general, female mitochondria demonstrate greater oxidative phosphorylation efficiency, enhanced antioxidant capacity and improved mitochondrial quality control compared with males. These differences are largely driven by sex hormones—particularly estrogens—which promote mitochondrial biogenesis through activation of PGC-1α, NRF1/2, and TFAM, enhance electron transport chain efficiency, and reduce reactive oxygen species (ROS) generation via upregulation of antioxidant enzymes such as SOD2 and GPx. In contrast, male mitochondria tend to exhibit higher basal ROS production, reduced coupling efficiency, and greater vulnerability to mitochondrial DNA (mtDNA) damage under metabolic or inflammatory stress. Additionally, females display more robust mitophagy—a specialized form of cellular autophagy that is affected early in MASH—and mitochondrial dynamics favoring fusion over fission, contributing to mitochondrial resilience and delayed apoptotic priming, as demonstrated in the present study. These sex-dependent mitochondrial phenotypes have been implicated in differential susceptibility to metabolic dysfunction–associated diseases beyond steatohepatitis (MASH), including cardiovascular disease, neurodegeneration, and cancer, with males exhibiting earlier mitochondrial failure and apoptosis-resistant phenotypes, whereas females show heightened sensitivity to mitochondrial stress but more efficient damage resolution. Collectively, sex-specific mitochondrial regulation represents a fundamental biological variable shaping disease progression and therapeutic responsiveness.

The findings of this study should be interpreted considering several limitations. We focused exclusively on sex-specific hepatic responses to dietary modulation and did not assess senescence activity in extrahepatic organs that may influence liver pathology. Prior studies have reported increased senescence-associated β-galactosidase (SA-β-gal) expression in the pancreas [69] and elevated senescence-associated secretory phenotype (SASP) markers, including p16 and p21, in hepatocytes—findings that are consistent with our observations. Given that cellular senescence is a central driver of aging and chronic disease, our results underscore dietary composition as a critical determinant of metabolic liver health. In addition, we did not evaluate ATP1A1 signaling activity, which may differ despite comparable protein expression levels. Further studies are warranted to elucidate the molecular mechanisms by which dietary interventions regulate senescence and SASP profiles and to determine whether such modulation can prevent or reverse MASH progression.

## 4. Materials and Methods

### 4.1. Animal Model

Seven-week-old C57BL/6 male and female mice (The Jackson Laboratory, Farmington, CT, USA) were randomly assigned to two dietary groups (n = 5 per group/sex). One group was exposed to a *high-fat diet* (HFD = 36.0% fat and 35.7% carbohydrate, Bio-Serv, NJ, USA) with 55% fructose water ad libitum, while the other group was fed with *normal mouse chow* (NMC = 7.2% fat and 61.6% carbohydrate, Bio-Serv, Flemington, NJ, USA) to serve as a control. Metabolic compartments were determined 24–48 h before euthanizing the animals at 0, 12, 24, and 48 weeks (0 W, 12 W, 24 W, and 48 W, Figure S1). The C57BL/6 breed is highly susceptible to metabolic dysfunctions such as diet-induced obesity, type 2 diabetes, hyperglycemia, and hyperinsulinemia [70,71].

The mice were induced (pentobarbital 5 mg/kg TBW, IP) for laparotomy at study points. Blood was collected from the infra-hepatic inferior vena cava (IVC), followed by the removal of the liver. The livers were washed with 10 mL of PBS at RT and divided into two parts, half snap-frozen in liquid nitrogen to be stored at −80 °C and half fixed at 4 °C (10% formaldehyde). Plasma obtained from the drawn blood was treated and saved for further analysis. Labeled samples were stored at −80 °C until used. All animal studies were approved by the University Institutional Animal Care and Use Committee (IACUC), following the National Institutes of Health (NIH) Guide for Care and Use of Laboratory Animals.

### 4.2. Liver Metabolism Measurements

#### 4.2.1. Total Body Compartments

EchoMRI-100H (Echo-MRI; Houston, TX, USA) was used to analyze the total body fat, water, and lean mass of the mice, following the manufacturer’s instruction manual. Briefly, the mice were restrained in a tube fitted to their size and weight for no more than 10 min, while the machine analyzed their body composition under the uninterrupted supervision of the operator. They were then returned to their normal housing upon completion of this survival procedure.

#### 4.2.2. Oxidative Species and Non-Targeted Metabolites

Glutathione sp. (GSH and GSSG) and non-targeted metabolites were measured in plasma using LC-MS/MS. A detailed LC-MS/MS methodology is included in Supplementary Materials.

### 4.3. Western Blotting Analysis

The expression of proteins involved in cellular metabolism and oxidative stress response (SIRT7, mTOR, Grb2, and p53) was evaluated by Western blot (WB). Liver tissue lysates were homogenized in RIPA buffer (pH = 7.4), centrifuged (14,000 rpm/15 min/4 °C), and supernatants were separated by SDS-PAGE and transferred to Protran nitrocellulose membranes (Thermo Fisher Scientific, Waltham, MA, USA). Blocked membranes were incubated with primary antibodies for each protein and probed with the corresponding horseradish peroxidase (HRP)-conjugated secondary antibodies. Membranes were developed using a Pierce ECL kit (Thermo Fisher Scientific, Waltham, MA, USA) with the FluorChem M System (Minneapolis, MN, USA). The blots were quantified using ImageJ software V9.01 (Fiji, NIH, Bethesda, MD, USA), and the integrated density of each band was normalized against the housekeeping protein (α-Tubulin). The details of the antibodies used and their dilutions are listed in.

### 4.4. Liver Morphology

Formalin-fixed liver tissue was processed, paraffin-embedded, and sectioned to generate slides for histological and immunohistochemical analyses. Hematoxylin and eosin (H&E), Masson’s trichrome, TUNEL, and β-galactosidase staining were performed according to standard protocols (see Supplementary Materials). Images were digitally acquired using a Leica fixed-stage confocal microscope (Leica DM6000 CFS, Leica Microsystems Inc., Deerfield, IL, USA) or an EVOS imaging system. ImageJ v1.51u (NIH, Bethesda, MD, USA) was used to quantify positive cells as a percentage of total cells and to measure cell size. All image acquisition and analyses were performed by investigators blinded to the experimental group assignment.

The liver NAFLD activity score (NAS) was determined by grading five images at 20×/40× magnification on H&E-stained liver slides. Grading was carried out using standard grading criteria for macro-vesicular steatosis, micro-vesicular steatosis, inflammatory cell infiltrate, and cellular hypertrophy. Steatosis was graded as either macro- if the fat vacuoles displaced the nucleus, or micro- if there was no nuclear displacement, and scored as **0** = <5%, **1** = 5–33%, **2** = 34–66%, and **3** = >66%. Inflammatory foci were defined as an aggregate of more than 5 inflammatory cells and were scored as **0** (<0.5 foci), **1** (0.5–1.0 foci), **2** (1.0–2.0 foci), and **3** (>2.0 foci). Lastly, hepatocellular hypertrophy was defined as cellular enlargement greater than 1.5 times the average diameter of the hepatocyte measured in the control slides [72,73]. Five representative images at 40× magnification were captured for each liver section from the mice in all groups.

*Liver fibrosis* was determined by grading five images at 20×/40× magnification of trichrome-stained liver slides on a categorical scale [71]. Images were graded according to the following aggregate scores saved for analysis: **0**: None; **1**: Enlarged, fibrotic portal tracts; **2**: Peri-portal or portal-portal septa, but intact architecture; **3**: Fibrosis with architectural distortion, but no obvious cirrhosis; **4**: Probable or definitive cirrhosis with bridging fibrosis [72,73]. Thereafter, five representative images at 20×/40× magnification were taken of the stained liver slides for each mouse in all the groups.

*Hepatocellular senescence* was determined by grading five images at 20×/40× magnification of liver slides stained for β-galactosidase activity (Cell Signaling Technology #9860, Danvers, MA, USA) [74].

*Apoptotic activity* in liver tissue was assessed using the terminal deoxynucleotidyl transferase dUTP nick-end labeling (TUNEL) method. The Click-iT Plus TUNEL assay kit (Invitrogen by Thermo Fisher Scientific, Waltham, MA, USA) was utilized according to the manufacturer’s instructions. Five images of the stained slides were captured at 20×/40× magnification for each animal in each group. For the senescence and apoptosis evaluation, positive cells for the staining were counted and expressed as a percentage of the total number of cells counted.

### 4.5. Statistical Analysis

Results are shown as box–whisker plots. Data are presented as median (central line), first and third quartiles (bottom and top of boxes, respectively), and whiskers (extreme values) from independent biological experiments. Differences among groups were determined by analyses of variance (ANOVA), Tukey’s post hoc test, and *t*-test using GraphPad Prism version 9.5.1 (GraphPad Software, Inc., San Diego, CA, USA). Sample size estimates were informed by preliminary data showing an 80% mean difference (range: 60–90%) in apoptosis between the groups. Assuming Δ = 80%, with a statistical power (β) of 0.8 and a significance level (α) of 0.05, the minimum sample size is four per group. For non-parametric data, Kruskal–Wallis test with Benjamini–Hochberg correction was used, and group comparisons were performed using the chi-square (χ^2^) test. Principal component analysis (PCA) was conducted to detect metabolite differences among groups using R-lab (version 4.4.2) software. Statistical significance was set at *p* < 0.05. Statistical tests, sample sizes, and *p*-values are provided in the figure legends.

## 5. Conclusions

The present study provides strong evidence of sex-specific dimorphism in the early molecular determinants of MASH, particularly in the regulation of apoptosis, cellular senescence, and metabolic stress responses to HFD.

## Data Availability

The original contributions presented in this study are included in the article/Supplementary Materials. Further inquiries can be directed to the corresponding author.

## Supplementary Materials

The following supporting information can be downloaded at https://www.mdpi.com/article/10.3390/ijms27031501/s1. References [75,76,77,78] are cited in the Supplementary Materials.

## Abbreviations

D5W: Dextrose 5% in water; GSH: reduced glutathione, GSSG: oxidized glutathione; HCC: hepatocellular carcinoma; HFD: high-fat diet; MASH: metabolic dysfunction-associated steatohepatitis; MASLD: metabolic dysfunction-associated steatotic liver disease; LC-MS: liquid chromatography–mass spectrometry; MRI: magnetic resonance imaging; NMC: normal mouse chow; PCA: principal component analysis; Sa-β-Gal: senescence-associated β-galactosidase; SASP: senescence-associated secretory phenotype; WAT: white adipose tissue.
