# Supplementary material for: Sexual Dimorphism in the Initial Apoptotic Switch During MASH Progression in Mice
Source: Int J Mol Sci. 2026 Feb 3;27(3):1501. doi: 10.3390/ijms27031501 (PMC12898622; doi:10.3390/ijms27031501)

SUPPLEMENTAL MATERIAL

FIGURES

Fig. S1.

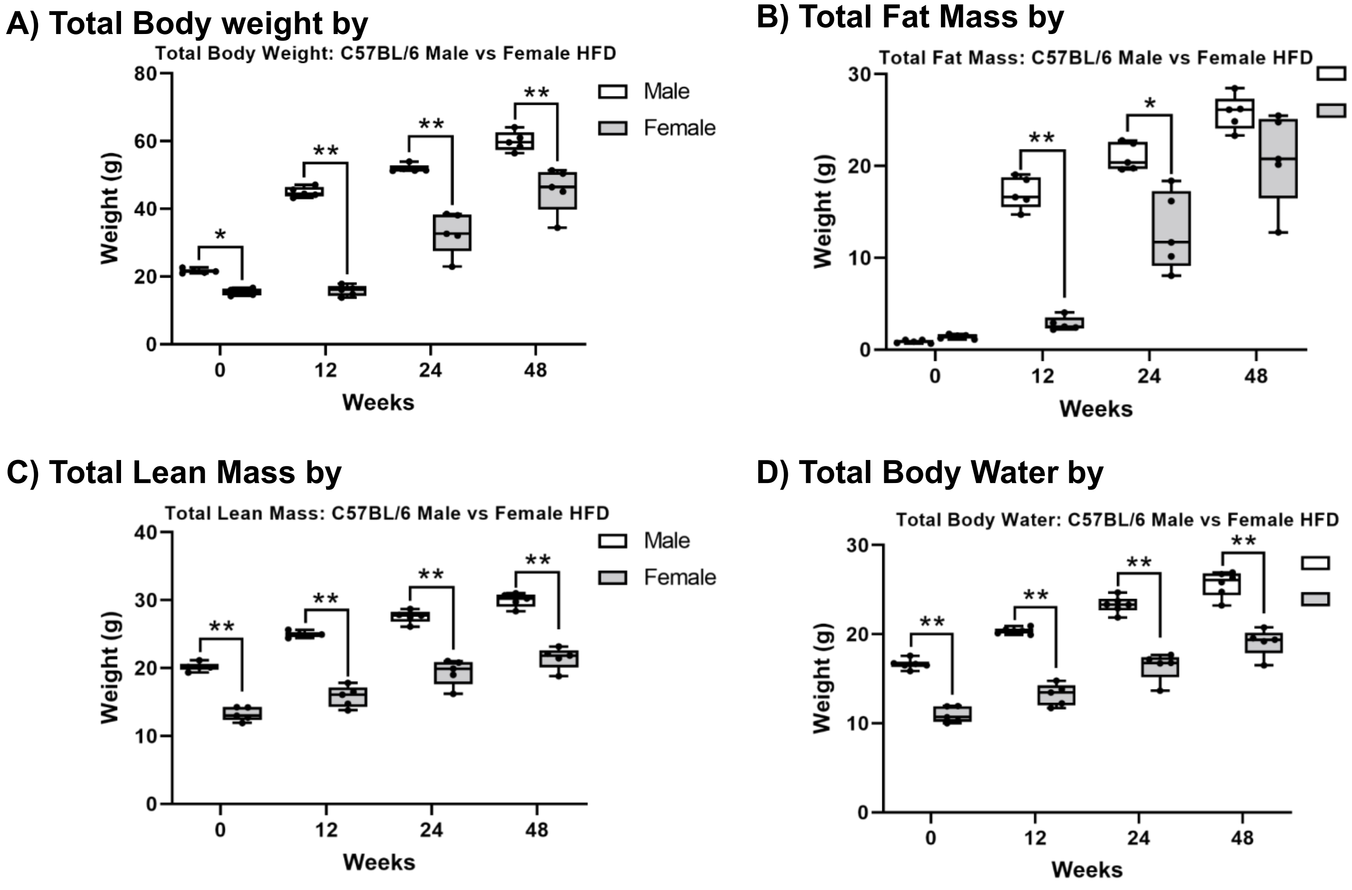

Fig S1. *Effect on total body compartments by sex in the diet induced MASH mice model.* **A)** Total body weight by gender: Mice exposed to HFD had a consistent increase in total body weight for both male and female groups. Male mice had higher weights than female mice. **B)** Total fat mass by gender: A significant increase in total fat mass was observed in the HFD for both males and females. at 12 & 24W, but by 48 weeks, there was no significant difference between the genders. **C)** Total lean mass by gender: Increased lean mass (a component of body composition calculated by subtracting body fat weight from total body weight) was observed for both males and females. **D)** Total body water by gender: There was a significant increase in total body water in males at all time points (* $p < 0.05$, ** $p < 0.01$, by ANOVA and Tukey's post hoc test/ t-test, n =5).

Fig. S2.

A) Glutathione sp. by diet

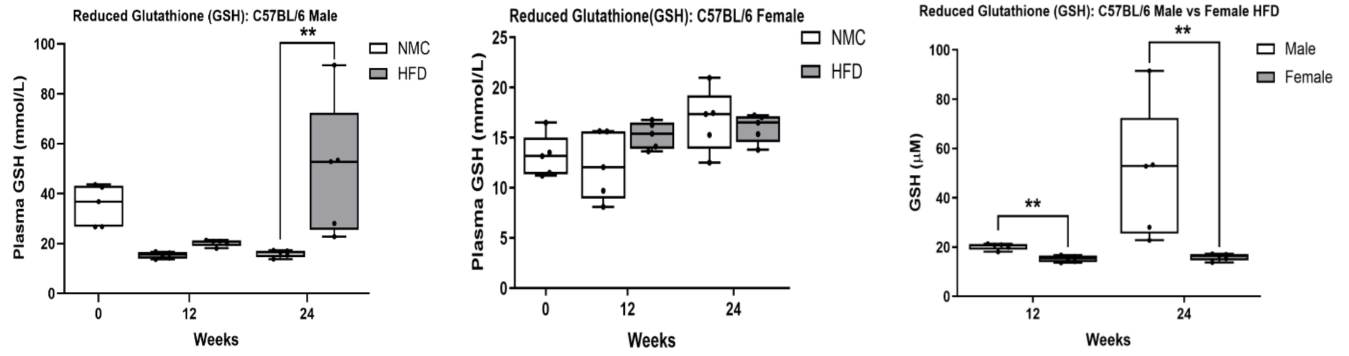

B) Glutathione sp. By sex in HFD

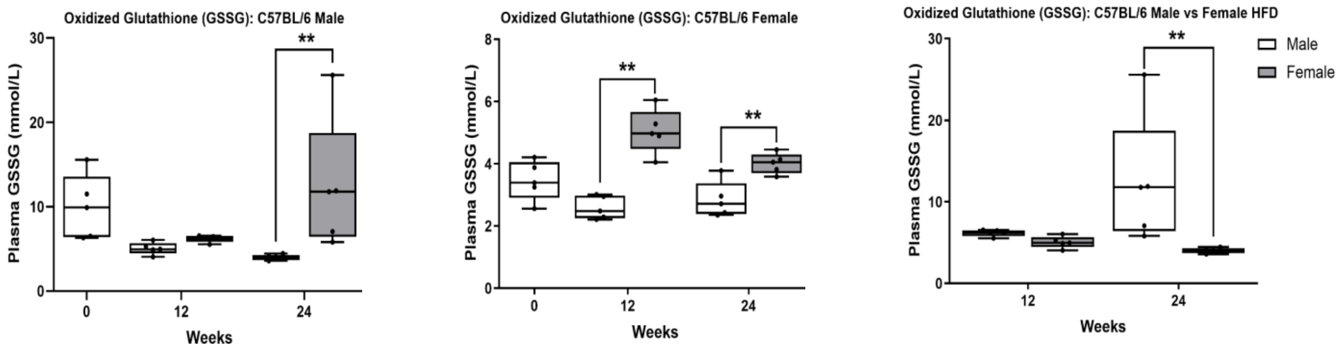

Fig S2. *Cellular redox assessment of MASH mice model by diet and sex.* **A)** There were significant differences in the Glutathione sp. (reduced glutathione (GSH), and oxidized glutathione (GSSG)) among groups and across experimental time points. There was an increase in reduced glutathione (GSH) levels in the HFD group *vs* NMC in males, but this was not observed in females. Males exhibited significantly higher GSH levels than females in the HFD group, and this trend was consistent at a 24-week time point. **B)** The level of oxidized glutathione (GSSG) was significantly higher in male groups on an HFD *vs* NMC group at 24 weeks, and a similar trend was observed in the female groups as well. At 0 weeks NMC, GSSG levels were also elevated in the male groups, but there were no significant differences between the male and female groups at 12 and 24 weeks in the NMC group. In contrast, males in the HFD group exhibited higher GSSG levels at 24 weeks when compared to the female HFD groups (* $p < 0.05$, ** $p < 0.01$, by ANOVA and Tukey's post hoc test/t-test, n = 5).

Fig S3.

Ophthalmate by diet and sex

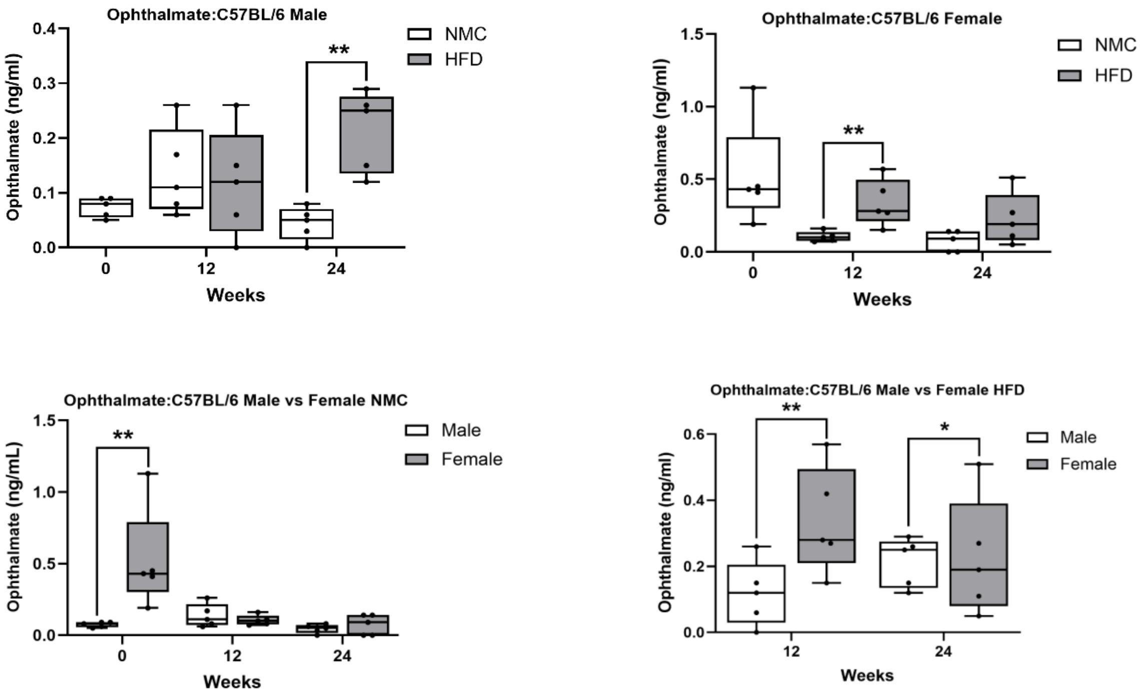

Fig S3. At 24 weeks, ophthalmate levels were higher in the male/female HFD *vs* NMC group. However, at 12W a difference was observed specifically in the female groups by diet. Male-NMC had lower levels of ophthalmate than female-NMC at 0W, and male-HFD had lower levels of ophthalmate than female-HFD at 12 & 24W. (* $p < 0.05$, ** $p < 0.01$, by ANOVA and Tukey’s post hoc test/t-test, $n = 5$).

Fig S4.

A) Plasma glucose by diet and sex

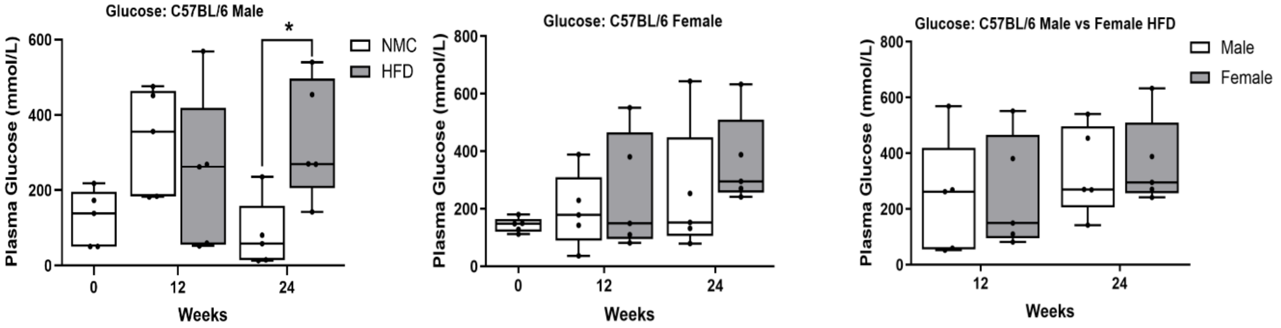

B) Plasma Lactate by diet and sex

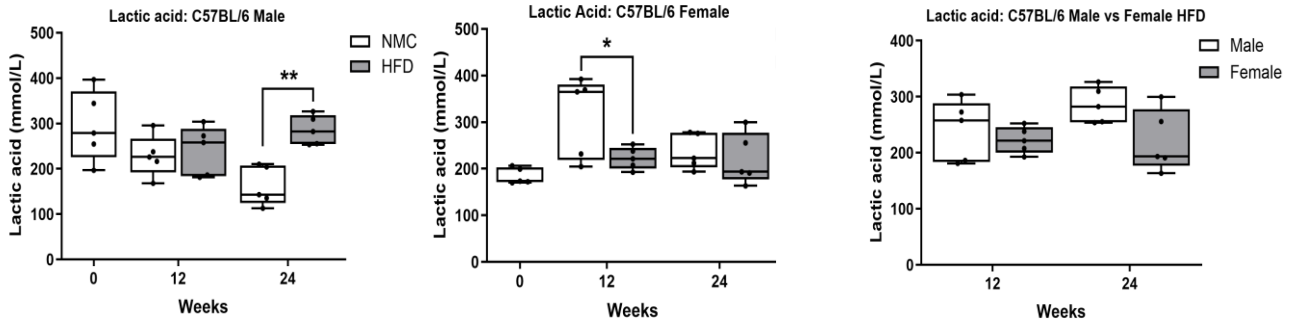

Fig S4. *Plasma Glucose and Lactate levels by sex and diet.* **A)** In mice fed a long-term HFD, males showed significantly higher glucose levels at 24 weeks than those on a NMC diet. **B).** Lactic acid: In mice fed a long-term HFD, male groups exhibited significantly higher levels of lactic acid at 24 weeks compared to those on a NMC diet. In contrast, among females, a significant difference in lactic acid levels between the HFD and NMC groups was observed at 12 weeks, but this difference was no longer evident at 24 weeks. (* $p < 0.05$, ** $p < 0.01$, by ANOVA and Tukey's post hoc test/t-test, n = 5).

Fig S5.

Plasma butyrate by diet and sex

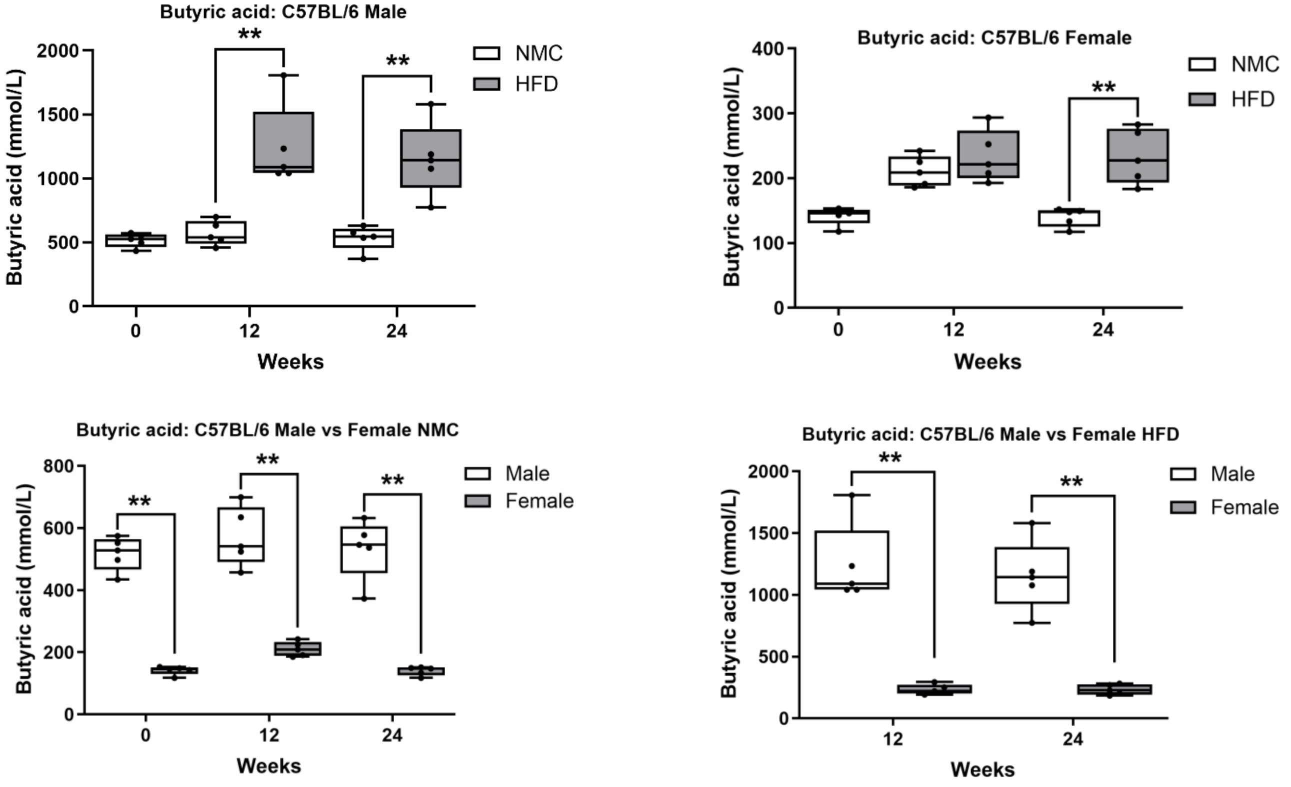

Fig S5. *Plasma butyrate by sex and diet in the MASH mice model.* Butyric acid: a significant difference in butyric acid in the HFD groups of both male and female groups at all-time points. Compared to the female groups, the male NMC and HFD groups showed a significantly higher butyric acid level (* $p < 0.05$, ** $p < 0.01$, by ANOVA and Tukey's post hoc test/t-test, n = 5).

Fig S6.

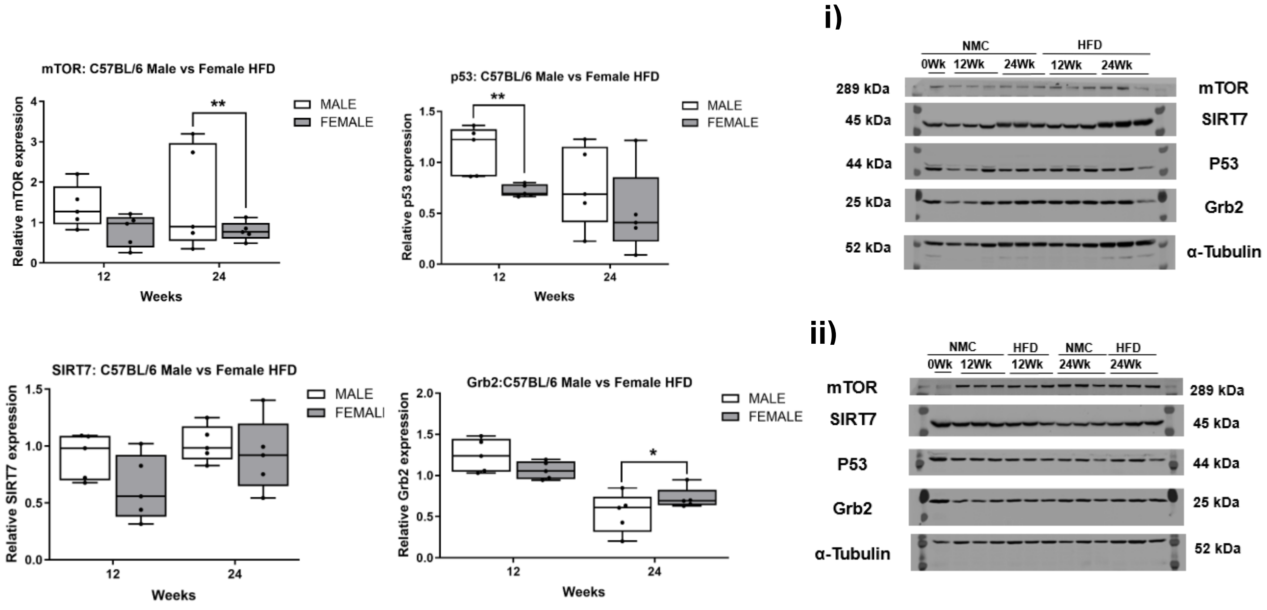

Fig S6. *Sex-specific expression of mitochondrial and apoptotic signaling regulators in the liver of C57BL/6J mice under high-fat diet-induced metabolic stress.* Representative Western blots show hepatic protein levels in (i) male and (ii) female mice at 0, 12, and 24 weeks. Male mice: In males (i), mTOR1 protein expression increased progressively on HFD, suggesting maximum levels at 24 weeks. SIRT7 levels remained comparatively stable in NMC and HFD groups at all-time points. p53 expression was not significantly different between diets or time points. Grb2 fluctuated little but did not show consistent trends with diet or time. Female: (ii) mTOR1 protein expression gradually decreased under HFD, especially at 24 weeks. SIRT7 expression dropped significantly under HFD at later stages. p53 expression rose notably after HFD at 24 weeks compared to NMC controls. Grb2 expression showed minimal time-dependent changes, with no significant diet-related effects in females. Sex Comparisons: (iii) The responses to the HFD varied by sex. On NMC, there were no differences in protein levels between males and females at any time point. However, on HFD, mTOR1 and Grb2 levels were significantly higher in males compared to females at 24 weeks. SIRT7 expression did not show any significant sex differences. Conversely, p53 levels were higher in females at 24 weeks. Statistical comparisons were carried out using two-way ANOVA followed by Tukey's post hoc test or t-tests to evaluate the impacts of diet, time, and sex, with significance indicated as $^*p < 0.05$ and $^{**}p < 0.01$ compared to corresponding NMC controls.

Fig S7.

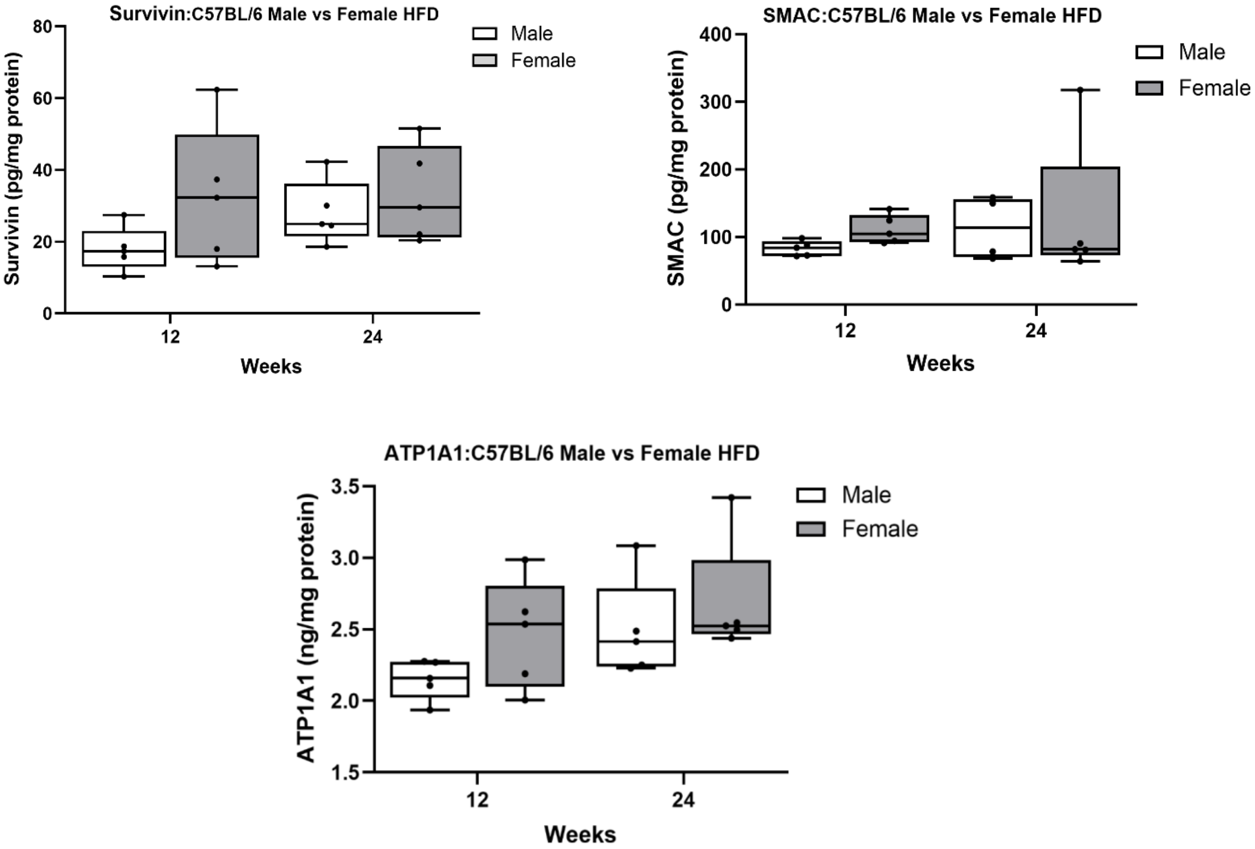

Fig S7. *Liver tissue concentration of survivin, SMAC and ATP1A1 in the MASH mice model.* There were no significant differences in liver tissue protein expressions in males vs. females on HFD at 12 and 24W ($p>0.05$).

Fig S8.

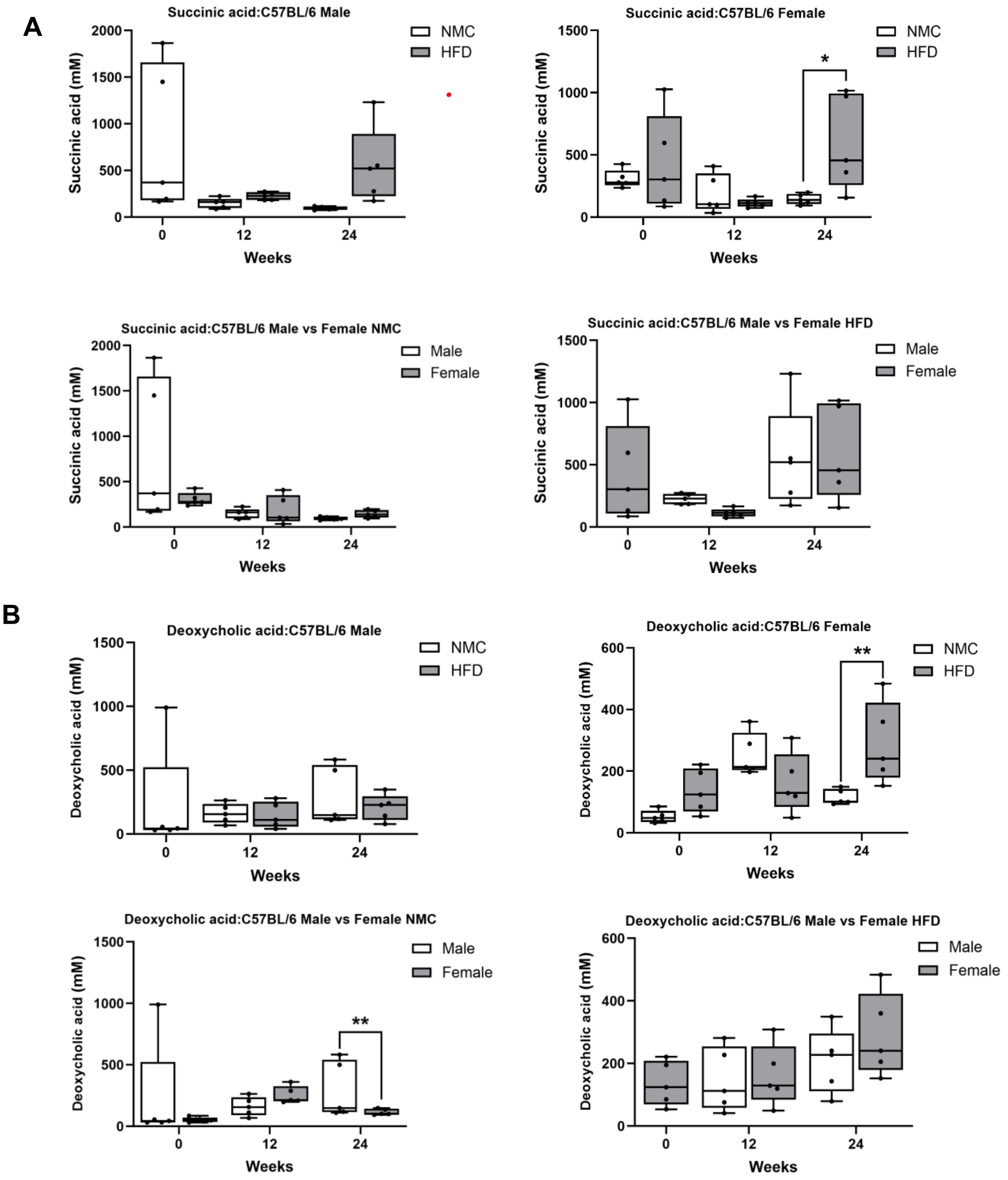

Fig S8. *Effect on non-targeted metabolites by diet and sex in MASH mice model.* **A)** HFD-induced female mice showed an increased level of succinic acid and deoxycholic acid at 24W. **B)** In the NMC groups, males exhibited higher levels of deoxycholic acid at 24 weeks (* $p < 0.05$, ** $p < 0.01$, by ANOVA and Tukey's post hoc test/t-test, n = 5).

EXPANDED METHODS.

<u>HPLC-MS/MS</u> *Plasma Treatment.* Heparinized blood was collected in glass tubes, gently inverted in an ice–water slurry for 1 min, and centrifuged at 3,000 × g for 10 min at 4 °C. To prevent oxidation, reduced glutathione (GSH) was immediately stabilized by derivatization with iodoacetate: 100 µL of whole blood was mixed with 100 µL of 50 mM iodoacetate in 10 mM ammonium bicarbonate buffer (pH 10, adjusted with concentrated ammonium hydroxide). After removal of the buffy coat and red blood cell pellet, plasma aliquots were treated with 50 µL of the iodoacetate buffer (1:1, vol:vol), transferred into pre-labeled microtubes, and rapidly frozen at −80 °C until analysis.

Liquid Chromatography–Mass Spectrometry (LC–MS) for Glutathione Species and Glucose. Plasma concentrations of reduced (GSH) and oxidized (GSSG) glutathione were quantified using previously validated LC–MS/MS methods with minor modifications. [(1–4)] Briefly, plasma samples were first treated with iodoacetate to derivatize GSH as GS-carboxymethyl, followed by dithiothreitol reduction to convert GSSG to GS-cyanomethyl using iodoacetonitrile. Homo-glutathione was spiked at the start of the assay as an internal standard. Calibration curves consisted of two blanks and seven calibration points, with ranges as follows: GSH, 0.78–200 µM; GSSG, 0.157–40 µM. A weighting factor of $1/x^2$ was applied to all calibration curves. Peak area ratios of analyte/internal standard were plotted against analyte concentrations. Electrospray ionization mass spectrometry (ESI–MS) of the derivatized products was performed on a Thermo Scientific TSQ Quantum Ultra mass spectrometer (Thermo Fisher Scientific, Waltham, MA) equipped with a heated electrospray ionization source (HESI-II) and coupled to an Agilent 1200 HPLC system. Chromatographic separation was achieved on a reversed-phase C18 column (Synergi 4 µ Hydro-RP, 50 × 2.0 mm, Phenomenex) using 1% acetonitrile containing 0.1% formic acid as the mobile phase at a flow rate of 0.1 mL/min (isocratic). Mass spectrometry was performed in positive ionization mode with the following parameters: ion spray voltage, 4000 V; ion transfer tube temperature, 400 °C; vaporizer temperature, 40 °C; sheath, auxiliary, and ion sweep gas settings at 50, 2, and 0 arbitrary units, respectively; argon collision gas pressure, 1.5 mTorr; collision energy, 15 V; peak width (Q1 and Q3), 0.7 FWHM. Data acquisition and analysis were conducted using Xcalibur software (version 2.1.0, Thermo Fisher Scientific). The area under the curve for each analyte was used to calculate concentrations. Glucose concentrations were measured using the same LC–MS procedure.

Liquid Chromatography–Mass Spectrometry (LC–MS) for Non-Targeted Metabolomics. Non-targeted metabolomics analysis was performed using thawed plasma samples subjected to solvent extraction with MAA (methanol: acetonitrile: acetone; 1:1:1). Internal standards were reconstituted in methanol:H_2O (2:98). Chromatographic separation was carried out on a 1290 Infinity Binary LC system (Agilent Technologies) equipped with a Waters Acquity UPLC HSS T3 column (1.8 µm, 2.1 × 100 mm) and a matching pre-column. The column was maintained at 55 °C with a flow rate of 0.45 mL/min. Total run time was 34 min, consisting of 7 min for system equilibration and 27 min for data acquisition.The mobile phases were as follows: **Phase A:** 0.1% formic acid in water; **Phase B:** 0.1% formic acid in methanol. The elution profile was: <u>0–20 min</u>: 98% A / 2% B → 20. <u>1–22 min</u>: 25% A / 75% B → 22. <u>1–30 min</u>: 2% A / 98% B → 30. <u>1–37 min</u>: re-equilibration to 98% A / 2% B. Positive and negative ion mass spectra were acquired in scan mode over a mass range of 50–1000 m/z. In-line mass calibration was performed using the following standards: **Positive mode:** debrisoquine sulfate (m/z 176.1182) and HP-0921 (m/z 922.0098, Agilent); **Negative mode:** 4-NBA (m/z 166.0146) and HP-0921 (m/z 966.0007, formate adduct, Agilent). The mass spectrometer parameters were as follows: ion source temperature, 325 °C; drying gas flow, 10 L/min; nebulizer pressure, 45 psi; sheath gas temperature, 400 °C; sheath gas flow, 12 L/min; capillary voltage, 4000 V; fragmentor voltage, 140 V; skimmer voltage, 65 V. Raw data were processed using the National Institute of Standards and Technology (NIST)

Automated Mass Spectral Deconvolution and Identification Software (AMDIS). From approximately 800 detected signals, 94 were consistently identified across 89% of all samples using both the in-house metabolomics library and the Fiehn library (Agilent Technologies, Santa Clara, CA). Data was further processed through the University Core Metabolomics Server. Metabolite concentrations were expressed as relative peak areas normalized to the corresponding internal standard in the same chromatogram. Certain small molecules—including glycerol, pyruvate, and acetoacetone—were targeted but not reliably detected in this model. All 91 identified metabolites were included in subsequent statistical analyses.

Non-target metabolomics in plasma includes the following lipids, amino-acids and carbo-hydrates (in alphabetical order): 16:0 LYSO PC (M+Cl)-; 16:0-18:0 PC (M+Cl)-; 18:0 LYSO-PE (M-H)-; 20:0 LYSO PC (M+Cl)-; 24:0 SM (D18:1/24:0) (M+Cl)-; 2-HYDROXY-3-METHYLBUTYRIC ACID (M-H)-; 2-HYDROXYBUTYRIC ACID (M-H)-; 3-(4-HYDROXYPHENYL)LACTIC ACID (M-H)-; 3'-CMP (M-H)-; 3-HYDROXY-3-METHYLGLUTARIC ACID (M-H)-; 3-METHYL-2-OXOPENTANOIC ACID (M-H)-; 3-UREIDOPROPIONIC ACID (M-H)-; 4-COUMARIC ACID (M-H)-; 4-HYDROXYBENZOIC ACID (M-H)-; 4-METHYL-2-OXOPENTANOIC ACID (M-H)-; ADIPIC ACID (M-H)-; ARACHIDIC ACID (M-H)-; ARACHIDONIC ACID (M-H)-; BEHENIC ACID (M-H)-; BENZOIC ACID (M-H)-; CAPRYLIC ACID (M-H)-; CIS-11-EICOSENOIC ACID (M-H)-; CITRAMALIC ACID (M-H)-; CITRIC ACID (M-H)-; CORTICOSTERONE (M+Cl)-; DEHYDROASCORBIC ACID (M-H)-; DEOXYCHOLIC ACID (2M-H)-; DEOXYCHOLIC ACID (M+Cl)-; DEOXYCHOLIC ACID (M-H)- ; DEOXYURIDINE (M+Cl)-; DEOXYURIDINE (M-H)-; D-GLUCOSAMINE 6-SULFATE (M-H)-[-H2O]; DOCOSAHEXAENOIC ACID (M-H)-; DUMP (M-H)-; EPIBRASSINOLIDE [ISTD] (M+Cl)-; ERUCIC ACID (M-H)-; FLAVIN ADENINE DINUCLEOTIDE (M-H)-; GLUCOSE (M+Cl)-; GLUTAMINE (M-H)-; GLUTARIC ACID (M-H)-; GLUTATHIONE (OXIDIZED) (M-H)-; HEPTADECANOIC ACID (M-H)-; HIPPURIC ACID (M-H)-; HOMOVANILLIC ACID (2M-H)-; HYODEOXYCHOLIC ACID (M-H)-; INDOLE-3-PYRUVIC ACID (M-H)-; INOSINE (M+Cl)-; INOSINE (M-H)-; INOSINE 5'-DIPHOSPHATE (M-H)-; ISOCITRIC ACID (M-H)-; ISOLEUCINE (M-H)-; KYNURENIC ACID (M-H)-; L-HISTIDINE (M-H)-; LIGNOCERIC ACID (M-H)-; LINOLEIC ACID (M-H)-; L-TRYPTOPHAN-15N2 [ISTD] (M-H)-; LYSINE (M-H)-; MALEIC ACID (M-H)-; MALIC ACID (M-H)-; METHYL BETA-D-GALACTOSIDE (M+Cl)-; METHYLMALONIC ACID (M-H)-; MYRISTIC ACID (M-H)-; MYRISTOLEIC ACID (M-H)-; N-ACETYLGLYCINE (M-H)-; N-ACETYL-L-ALANINE (M-H)-; N-ACETYL-L-LEUCINE (M-H)-; N-ACETYL-L-PHENYLALANINE (M-H)-; NERVONIC ACID (M-H)-; OLEIC ACID (M-H)-; OXALOACETIC ACID (M-H)-[-H2O]; PALMITIC ACID (M-H)-; PALMITOLEIC ACID (M-H)-; PANTOTHENIC ACID (M+Cl)-; PANTOTHENIC ACID (M-H)-; PHENYLALANINE (M-H)-; PHTHALIC ACID (M-H)-; PIMELIC ACID (M-H)-; SEBACIC ACID (M-H)-; STEARIC ACID (M-H)-; SUCCINIC ACID (M-H)-; TAURINE (M-H)-; THYMIDINE (M+Cl)-; THYMIDINE (M-H)-; THYMINE-D4(METHYL-D3,6-D1) [ISTD] (M-H)-; TRANS-ACONITIC ACID (M-H)-; TYROSINE (M-H)-; URIC ACID (M-H)-; URIDINE (M+Cl)-; URIDINE (M-H)-; XANTHINE (M-H)-; XANTHOSINE (M-H)-; ZEATIN [ISTD] (M+Cl)-; ZEATIN [ISTD] (M-H)-.

Hematoxylin and Eosin (H&E) Staining. Liver sections were deparaffinized, rehydrated, and stained following standard protocols. Briefly, slides were immersed in filtered Harris hematoxylin for 2 min, rinsed with water, and differentiated in 0.3% ammonium hydroxide (10–20 dips). After rinsing with water, slides were counterstained in Eosin-Y (10 dips), washed, and dehydrated sequentially in graded ethanol solutions: 70% ethanol (1 min), 80% ethanol (1 min), 95% ethanol (1 min, three times), and 100% ethanol (1 min, three times). Finally, slides were cleared in xylene (1 min, twice) and cover slipped using Permount mounting medium.

Masson's Trichrome Staining. Following deparaffinization and rehydration, liver sections were immersed in 40 mL of Bouin's solution in a plastic Coplin jar with the lid applied loosely, microwaved on high for 30 seconds, and allowed to stand for 5 minutes. Slides were then rinsed in tap water for 5 minutes until the yellow color was completely removed. Sections were stained in Working Weigert's Iron Hematoxylin solution for 10 minutes, rinsed in tap water for 10 minutes, and then incubated in Masson's Trichrome stain for 15 minutes. Slides were briefly immersed in 1% acetic acid solution for 1 minute, rinsed in deionized water for 30 minutes, and dehydrated through two changes of 100% ethanol (1 minute each). Clearing was performed with three changes of xylene (1 minute each). Finally, slides were cover slipped using Permount mounting medium and stored overnight at room temperature to allow the medium to set prior to imaging.

References

1. Andres Ibarra R, Abbas R, Kombu RS, Zhang GF, Jacobs G, Lee Z, et al. Disturbances in the glutathione/ophthalmate redox buffer system in the woodchuck model of hepatitis virus-induced hepatocellular carcinoma. HPB Surg. 2011; 2011:789323.
2. Ibarra R, Dazard JE, Sandlers Y, Rehman F, Abbas R, Kombu R, et al. Metabolomic Analysisof Liver Tissue from the VX2 Rabbit Model of Secondary Liver Tumors. HPB Surg. 2014; 2014:310372.
3. Kombu RS, Zhang GF, Abbas R, Mieyal JJ, Anderson VE, Kelleher JK, et al. Dynamics of glutathione and ophthalmate traced with 2H-enriched body water in rats and humans. Am J Physiol Endocrinol Metab. 2009;297(1): E260-9.
4. Sanabria JR, Kombu RS, Zhang GF, Sandlers Y, Ai J, Ibarra RA, et al. Glutathione species and metabolomic prints in subjects with liver disease as biological markers for the detection of hepatocellular carcinoma. HPB (Oxford). 2016;18(12):979-90.

Wester Blots

FEMALE WESTERN BLOT RESULTS

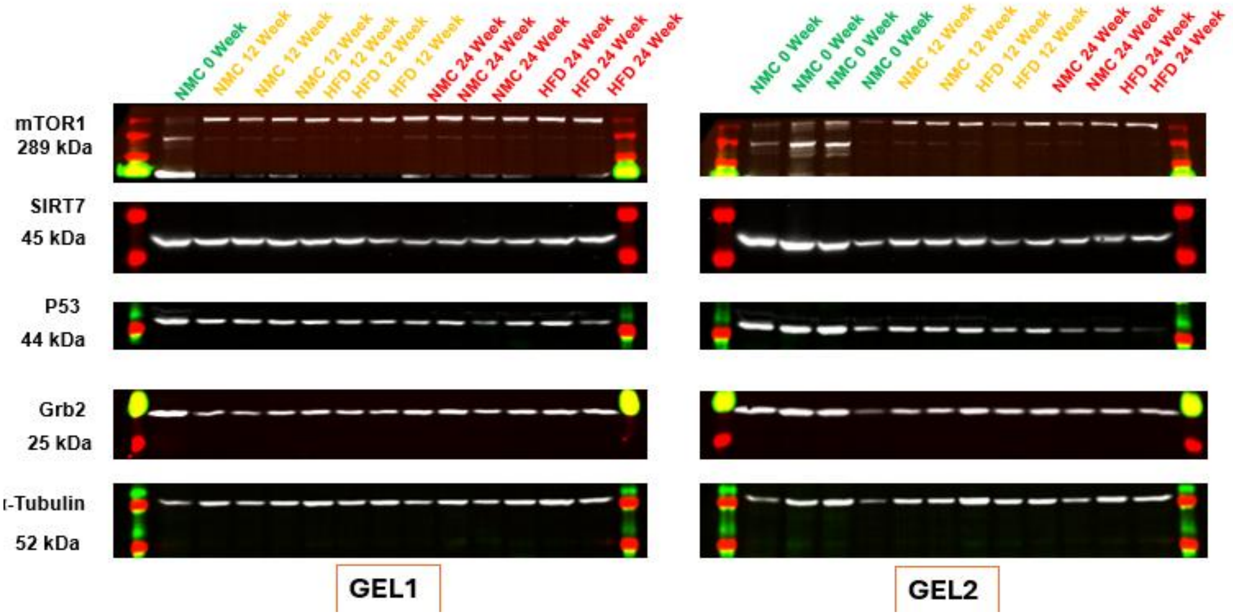

MALE WESTERN BLOT RESULTS

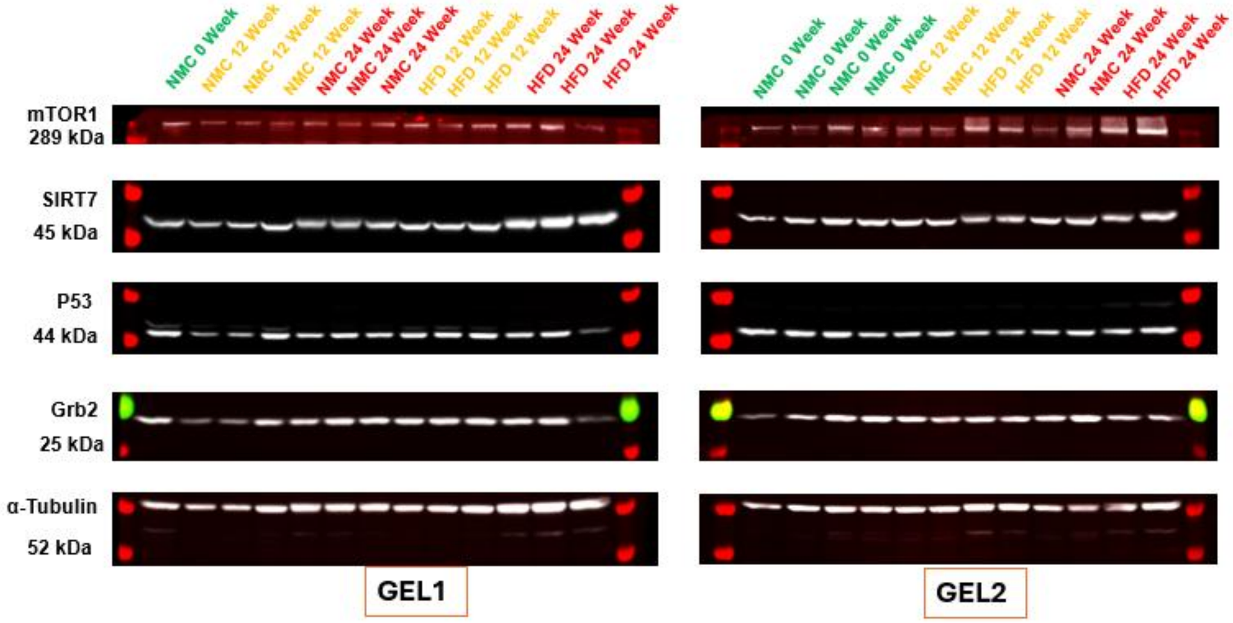

Supplement: Supplementary file 1 [file ijms-27-01501-s001.zip › ijms-4074497-supplementary.pdf]
